# Clinical Impact of a Germline CD19 Variant on Treatment Outcome After CAR-T Cell Therapy in Relapsed/Refractory Mantle Cell Lymphoma

**DOI:** 10.3390/cancers18132110

**Published:** 2026-06-29

**Authors:** Simona Andrea Ruckstuhl, Katja Seipel, Inna Shaforostova, Martina Bertschinger, Ulrike Bacher, Thomas Pabst

**Affiliations:** 1 Department of Medical Oncology, Inselspital, Bern University Hospital, University of Bern, 3010 Bern, Switzerland; simona.ruckstuhl@students.unibe.ch (S.A.R.); innaivanovna.shaforostova@insel.ch (I.S.); martina.bertschinger@insel.ch (M.B.); 2 Department for Biomedical Research (DBMR), University of Bern, 3008 Bern, Switzerland; veraulrike.bacher@insel.ch; 3 Department of Hematology, Inselspital, Bern University Hospital, University of Bern, 3010 Bern, Switzerland

**Keywords:** mantle cell lymphoma (MCL), chimeric antigen receptor (CAR), progression-free survival (PFS), overall survival (OS), single-nucleotide polymorphism (SNP)

## Abstract

Background: Brexu-cel is an established CD19-directed CAR-T cell therapy for relapsed or refractory mantle cell lymphoma, but clinical outcomes are heterogeneous, and germline determinants of response are poorly defined. Methods: We investigated the clinical impact of the *CD19* single-nucleotide polymorphism rs2904880 on treatment outcome after brexu-cel CAR-T cell therapy by analyzing genotype-outcome associations in a retrospective single-center cohort of patients with relapsed or refractory mantle cell lymphoma treated with this CAR-T cell therapy. Results: Overall survival varied according to *CD19* polymorphism, with improved survival of patients carrying the V174 homozygous genotype compared with L174V heterozygotes. Conclusions: The germline *CD19* polymorphism may have implications for future risk stratification after brexu-cel CAR-T cell therapy in mantle cell lymphoma, but requires validation in larger, multicenter cohorts.

## Simple Summary

Despite therapeutic advances, mantle cell lymphoma remains an incurable disease. CD19-directed CAR-T cell therapy can induce durable responses in patients with relapsed or refractory mantle cell lymphoma, but outcomes vary considerably. Germline variants of the *CD19* gene may contribute to a heterogeneous response. In this study, we examined a common genetic variant in the *CD19* gene in mantle cell lymphoma patients receiving brexu-cel, where the *CD19* rs2904880 V174 homozygous genotype was associated with longer overall survival. This *CD19* polymorphism may be relevant for future risk stratification in CD19-directed CAR-T cell therapy, but needs to be confirmed in larger, multicenter studies.

## 1. Introduction

Mantle cell lymphoma (MCL) is a rare subtype of aggressive non-Hodgkin’s lymphoma (NHL), accounting for approximately 3–10% of all NHL cases [1,2]. In the current classifications of lymphoid neoplasms, including the current World Health Organization (WHO) and the International Consensus Classification (ICC) systems, MCL is recognized as a distinct mature B-cell lymphoma with characteristic clinical and biological features [3,4]. It is characterized by a broad clinical spectrum, ranging from indolent or even asymptomatic courses to clearly aggressive disease with an unfavorable prognosis [1,5]. MCL predominantly affects older adults, with a median age at initial diagnosis between 60 and 70 years and shows a male predominance [2]. Despite recent therapeutic advances, including targeted agents and immune- and cellular-based approaches, MCL remains associated with a poor overall prognosis and is largely considered incurable [6,7,8]. Over recent years, the management of relapsed of refractory MCL (r/r MCL) has shifted from conventional chemo-immunotherapy towards covalent Bruton’s tyrosine kinase (BTK) inhibitors and, more recently CD19-directed CAR-T cell therapy [8,9,10]. Real-world data from the pre-CAR-T era showed that high-dose chemo-immunotherapy followed by autologous stem cell transplantation (ASCT) still led to relapse in 44% of transplanted patients and a median PFS of only 28 months after post-ASCT relapse, illustrating the limited long-term efficacy of this approach [11].

Chimeric antigen receptor (CAR)-T cell therapy is an adoptive cellular immunotherapy in which autologous T cells are collected and genetically modified to express a synthetic chimeric antigen receptor (CAR) targeting a defined surface antigen such as CD19, thereby enabling antigen-specific recognition and killing of malignant B-cells [12,13,14]. CAR-T cell therapy has therefore emerged as an important treatment option for r/r MCL, with brexucabtagene autoleucel (brexu-cel) being the first CD19-directed CAR-T cell product approved for this indication [15,16,17,18] and widely regarded as a practice-changing option that has substantially improved outcomes for patients with r/r MCL, particularly after BTK inhibitor failure [8,12,19]. Real-world studies from the United States and Europe have confirmed the efficacy of brexu-cel in r/r MCL, consistently reporting overall response rates of approximately 80–95% and complete response rates of around 60–85% [17,20,21,22,23], with similar results even in heavily pretreated, predominantly older patients [24]. However, despite the high response rates achieved with brexu-cel, outcomes after CAR-T cell therapy in r/r MCL remain heterogenous and a considerable proportion of patients either fails to achieve a durable complete remission or ultimately experiences disease progression [15,17,20,21]. This clinical heterogeneity highlights the need to better understand biological determinants of response to CD19-directed CAR-T cell therapy. To date, however, robust clinical or molecular predictors of response and toxicity to brexu-cel in r/r MCL are lacking [22,25].

A common germline single-nucleotide polymorphism (SNP) in CD19, rs2904880, which encodes either leucine or valine at position 174, has recently been associated with differential outcomes after CD19-directed CAR-T cell therapy in diffuse large B-cell lymphoma (DLBCL) [26]. As residue 174 lies within the extracellular domain of CD19 in proximity to critical regions for antibody recognition, the L174V substitution may subtly alter epitope presentation and thereby modulate CD19-directed CAR-T cell binding and activation [26]. Whether this CD19 polymorphism also modulates clinical outcomes and toxicity of CD19 CAR-T cell therapy in patients with r/r MCL, however, remains unknown. Against this background, we hypothesized that the CD19 germline polymorphism rs2904880 may modulate clinical outcomes in patients with r/r MCL treated with brexu-cel.

The aim of this study was to investigate the clinical impact of the CD19 rs2904880 in r/r MCL patients undergoing treatment with brexu-cel.

## 2. Patients and Methods

### 2.1. Study Design and Patient Cohort

This retrospective, single-center observational study was conducted at the Bern University Hospital, Bern, Switzerland, and included patients treated between 23 February 2020 and 10 January 2025. Eligible patients were adults (≥18 years) with histologically confirmed mantle cell lymphoma and documented relapse after at least one prior systemic therapy. The study was conducted according to the guidelines of the Declaration of Helsinki and approved by decisions of the local ethics committee of Bern, Switzerland, decision number 2025-00853, date of approval 24 April 2025.

### 2.2. CD19 Gene Analysis

Peripheral blood mononuclear cells (PMBCs) were obtained prior to CAR-T cell infusion, from which genomic DNA was subsequently isolated. *CD19* exons were amplified using FIREPol DNA polymerase (Solis Biodyne, Tartu, Estonia) and gene-specific primers (forward: 5′-CTCCCTCTCCTGGGTGTCTCTGCA-3′; reverse: 5′-CCCAGTACCCCCACA GATGCCT-3′). Sanger sequencing of the PCR products was carried out by Microsynth, Balgach, Switzerland.

### 2.3. Study Endpoints

The primary endpoint of this study was the clinical outcome including progression-free survival (PFS) and overall survival (OS). Secondary endpoints included the association with patients’ baseline clinical characteristics, disease-specific features and therapy-associated toxicities. The grading criteria of the American Society for Transplantation and Cellular Therapy (ASTCT) were used to assess and grade cytokine release syndrome (CRS) and immune-effector cell-associated neurotoxicity syndrome (ICANS).

### 2.4. Statistical Analysis

Overall survival (OS) was defined as the interval from CAR-T cell infusion until death from any cause. Progression-free survival (PFS) was defined as the interval from CAR-T cell infusion to either relapse or death. Patients who had not experienced an event at the time of analysis were censored at their last documented follow-up. Statistical analyses were performed using GraphPad Prism 10 ^®^. Categorical variables were compared using Fisher’s exact test. For continuous variables, non-parametric approaches were applied: the Mann–Whitney U test was used for two-group comparisons and the Kruskal–Wallis test was used when three independent groups were analyzed. PFS and OS curves were estimated using the Kaplan–Meier method and curves were compared using the log-rank (Mantel–Cox) test as the primary method, as it is the standard approach and equally weights events over the entire follow-up period. In addition, we calculated the log-rank test for trend and the Gehan–Breslow–Wilcoxon test as pre-specified secondary, exploratory analysis with different weighting schemes to events over time. Subgroups with a sample size of *n* = 1 were reported descriptively only and were not included in the inferential statistical analyses. Missing data were presented descriptively in a separate category but were excluded from inferential statistical analyses. Statistical significance was defined as a *p* value < 0.05 and percentage results were rounded to whole numbers.

## 3. Results

### 3.1. Prevalence of the CD19 L174 Allele

The sequence of the *CD19* gene spanning exons 3 and 4 was determined in the peripheral blood of 21 MCL patients evaluated for CAR-T cell therapy at our center. A total of 13 patients (62%) were homozygous for the *CD19* rs2904880 V174 allele (V174hom), 8 patients (38%) were heterozygous (L174Vhet), corresponding to a minor allele frequency (MAF) of 0.19. The *CD19* rs2904880 allele frequencies reported in the global population vary according to the subpopulation with lower frequencies in African and Asian populations (MAF 0.05–0.18), and higher frequencies in European populations (MAF 0.28–0.34). The global minor allele frequency is indicated at MAF 0.23 (TOPMED, a sample size of 264,690, access date 1 June 2026 http://www.ncbi.nlm.nih.gov/snp/docs/gsr/alfa/).

### 3.2. Clinical Characteristics at Baseline

In total, 21 MCL patients received FMC63-anti-CD19-CAR-T cell therapy (brexucabtagene autoleucel). The median age at initial diagnosis was 67 years, with a male predominance (Table 1). The majority had a classical cytomorphological presentation and disease stage IV according to the Ann-Arbor classification system. Nearly half of the patients (48%) had received more than three prior lines of treatment before CAR-T cell therapy. All three cases with CNS involvement were in the V174hom group. Across all comparisons, no statistically significant differences between genotype groups were observed.

### 3.3. Treatment Characteristics

The CAR-T treatment details were evaluated for the overall cohort, as well as separately within the two genetic subgroups (Table 2). The cohort had a median age of 73 years at CAR-T cell infusion and the median interval from diagnosis to CAR-T therapy was four years. The majority of patients presented with stable or progressive disease at the time of CAR-T treatment (38% and 33%, respectively). Overall, 48% of the patients received bridging chemotherapy and 5% received bridging radiotherapy. All patients underwent lymphodepleting chemotherapy, comprising either fludarabine and cyclophosphamide or bendamustine. The median lactate dehydrogenase (LDH) level prior to CAR-T cell infusion was 210 U/L and 8 patients (42%) had an elevated LDH (>250 U/L). All patients were treated with brexucabtagene autoleucel (brexu-cel, Tecartus ©). No statistically significant differences between genotype subgroups were observed.

### 3.4. Early Therapy-Associated Toxicities

Early outcomes, including CAR-T cell-associated toxicities, were evaluated for the overall cohort and separately for the two genetic subgroups (Table 3). Cytokine release syndrome (CRS) was observed in 76% of patients with a median onset of 5 days after CAR-T cell infusion. Severe CRS (Grade 3) was seen in 5% of cases. Immune effector cell-associated neurotoxicity syndrome (ICANS) was observed in 48% of patients with a median onset of 10 days after infusion. Of these, 19% of cases were classified as severe ICANS (Grade 3–4). Digital droplet PCR demonstrated peak plasma levels of the CAR-T cell product at a median of 13 days after infusion with a median of 7237 copies per microgram of cell-free DNA. The inflammatory markers reached their maximum levels earlier: C-reactive protein (CRP), interleukin-6 (IL-6) and serum ferritin peaked at a median of 6, 8 and 11 days after infusion, respectively. The median peak levels were 56 mg/L, 638 pg/mL and 1474 ug/L, respectively. There were no statistically significant differences in these parameters between the genotype subgroups.

### 3.5. Treatment Outcomes

Treatment responses and clinical outcomes after CAR-T cell therapy were analyzed for the entire cohort and for the two genetic subgroups (Table 4). The overall response rate was 90%, with complete remission in 16 patients (76%). At the last follow-up, 13 patients (62%) were still in complete remission. Lymphoma-related events occurred in 4 patients, including three relapses after CR/PR and one case of primary refractory disease. Overall, 9 patients died, with four tumor-associated deaths, two therapy-associated deaths, one death related to a secondary malignancy and two deaths of other or unknown cause. No statistically significant differences between the genotype subgroups were observed.

**Figure 1:**
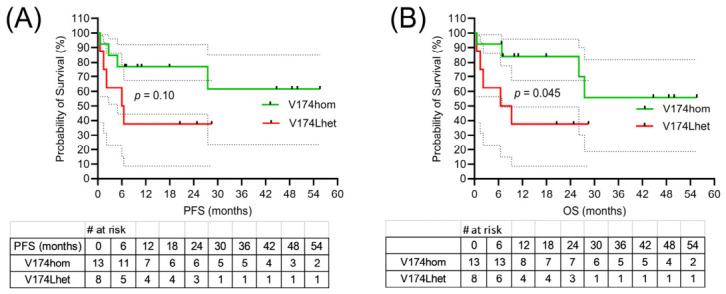
Survival outcomes after brexu-cel therapy according to *CD19* rs2904880 genotypes. Progression-free survival (**A**) and overall survival (**B**) according to *CD19* genotypes V174hom (green line) and V174Lhet (red line). The displayed *p*-values are based on the Gehan–Breslow–Wilcoxon test. The 95% confidence intervals are marked with gray dashed lines.

Treatment outcomes were evaluated for the overall cohort and for the *CD19* genetic subgroups (Figure 1). A trend toward longer survival was observed in the V174hom subgroup, with a median PFS and OS over four years compared with a median of six months in the L174Vhet subgroup. While the log-rank test did not reach statistical significance (*p* = 0.075), the Gehan–Breslow–Wilcoxon test indicated a significant difference in OS between the *CD19* genotype groups (*p* = 0.045). In contrast, the PFS did not differ significantly between the *CD19* genotype groups (log-rank test *p* = 0.12, Gehan–Breslow–Wilcoxon test *p* = 0.10).

Treatment outcomes were evaluated in a multivariate analysis with parameters of possible impact on the clinical outcome, including LDH levels, number of prior therapy lines and CRS. The multivariate analysis supported the trend observed in the univariate analysis for the CD19 major allele V174 to be a prognostic indicator for overall survival with an HR 0.08 at a *p*-value of 0.04 (Table 5). Elevated LDH levels were also predictive for outcomes with an HR of 63 at a *p*-value of 0.01, a higher number of prior therapy lines with an HR of 5.8 at a *p*-value of 0.15; and a CRS with an HR of 2.45 at a *p*-value of 0.48.

## 4. Discussion

In this retrospective analysis, we investigated the influence of the germline *CD19* gene single-nucleotide polymorphism rs2904880 on clinical characteristics and outcomes in patients with relapsed or refractory mantle cell lymphoma treated with brexu-cel. The allele frequencies observed in our cohort of mantle cell lymphoma patients were comparable to those reported in the global population, indicating no association of the *CD19* rs2904880 genotype with disease susceptibility. There was a statistically significant difference in the overall survival according to *CD19* genotype, suggesting that germline variation in the *CD19* gene may influence outcomes after CD19-directed CAR-T cell therapy in mantle cell lymphoma. All other evaluated variables, including baseline clinical characteristics and CAR-T cell-related toxicities, did not differ significantly between the genotype groups.

In the analysis of overall survival, only the Gehan–Breslow–Wilcoxon test reached statistical significance, whereas the log-rank test did not. Given that the Gehan–Breslow–Wilcoxon test assigns greater weight to earlier events, this pattern may indicate that any potential effect of the *CD19* polymorphism is concentrated in the early follow-up period. However, in view of the limited sample size and the discordant results between the two tests, this observation should be interpreted with caution. For the progression-free survival, neither statistical test showed a statistically significant difference in the *CD19* genotype subgroups. In this cohort, we therefore found no clear evidence that rs2904880 influences the risk of disease progression after CAR-T cell therapy, and these findings should be regarded as exploratory given the limited sample size and follow-up. A true lack of association between this polymorphism and progression risk cannot be excluded.

Consistent with a broader role of host genetics, recent work in large B-cell lymphoma has linked germline immune gene variants to variability in toxicity and efficacy of CD19-directed CAR-T cell therapy [27]. Building on this concept, a prior study in patients with diffuse large B-cell lymphoma investigating rs2904880 reported more favorable outcomes in carriers of the CD19 minor allele L174 after CD19-directed CAR-T cell therapy [26]. In contrast, in our cohort of MCL patients, we observed an advantage for the genotype V174hom over the L174Vhet genotype, indicating that patients without the L174 allele were associated with better overall survival. This inverse association pattern suggests that the impact of rs2904880 on CAR-T cell outcomes is context-dependent rather than uniform across different clinical settings.

One potential source of heterogeneity lies in the specific CD19-directed CAR-T cell products used in the respective cohorts. Currently, four CD19 CAR-T cell products are approved by the FDA for various B-cell lymphomas: axicabtagene ciloleucel (axi-cel), tisagenlecleucel (tisa-cel), lisocabtagene maraleucel (liso-cel) and brexucabtagene autoleucel (brexu-cel, KTE-X19) [28]. These products differ in their manufacturing processes, CAR constructs and cellular composition, including costimulatory domains and hinge regions [29]. Axi-cel and brexu-cel incorporate a CD28 costimulatory domain and use CD28 as a hinge domain, whereas tisa-cel and liso-cel use a 4-1BB costimulatory domain, with tisa-cel employing a CD8α hinge and liso-cel an IgG4 hinge. Consequently, axi-cel and brexu-cel share an identical CAR design with CD28-derived hinge, transmembrane and costimulatory domains [30]. The main technological distinction between the two products is an additional T cell enrichment step in brexu-cel manufacturing, which depletes circulating CD19-positive tumor cells from the leukapheresis product [12,19]. In the DLBCL cohort patients were treated with axi-cel and tisa-cel [26], whereas in this study MCL patients were treated with brexu-cel. Looking ahead, the development of dual-targeted CAR-T cell constructs, simultaneously targeting CD19 and CD20, is likely to broaden the spectrum of CD19-based therapies and may alter how product-related characteristics interact with target-antigen heterogeneity in the future. Although current data for such dual-target approaches come from early phase I/II studies only, they indicate a potential to evolve into future standards of care if confirmed in larger, multicenter trials [31]. Beyond such product-related differences, there is evidence that each B-cell malignancy exhibits a distinct biology that may differentially interact with CD19 CAR-T cells. Comparative analyses of outcomes with the same CD19 CAR products across multiple indications consistently show that efficacy, primary resistance, CD19-negative relapse and acute toxicities vary according to the underlying disease, supporting the view that entity-specific biology significantly shapes CAR-T cell performance [29]. Available clinical data further suggest that CD19-directed therapies can retain substantial activity even in cases with dim or undetectable CD19 expression and baseline CD19 status has not consistently predicted response to axi-cel, tisa-cel or brexu-cel [32]. Thus, it seems unlikely that the opposite genotype-outcome associations observed in DLBCL and MCL are driven solely by gross differences in CD19 antigen density. Since both our cohort and the PPM1D analysis [33] focus on patients with MCL, it is plausible that unique MCL-specific tumor biology—including its characteristic CCND1-driven cell-cycle deregulation, frequent DNA damage response pathway alterations and distinctive patterns of immune evasion involving checkpoint upregulation and micro-environmental reprogramming [34]–may influence how rs2904880-related changes in the CD19 epitope and product-specific CAR architectures translate into clinical benefit, potentially contributing to the observed opposite survival effects of the germline variant in MCL versus DLBCL. Since both our cohort and the PPM1D analysis [32] focus on patients with MCL, the observed effects may therefore reflect entity-specific features rather than a uniform class effect across all CD19 CAR-T cell-treated lymphomas. In addition, patient- and disease-related factors such as age, comorbidities and tumor burden may further modulate outcomes after CD19 CAR-T cell therapy. Taken together, the opposite effects of rs2904880 observed in MCL compared with the DLBCL study may arise from complex interactions between entity-specific tumor biology, product-specific characteristics and individual patient factors. A chance finding cannot be excluded given the limited sample size. Beyond germline *CD19* variation, additional tumor-intrinsic and micro-environmental mechanisms are also likely to contribute to response heterogeneity after CD19-directed CAR-T cell therapy in MCL [12,33,34], but these were not addressed in our study.

**Figure 2:**
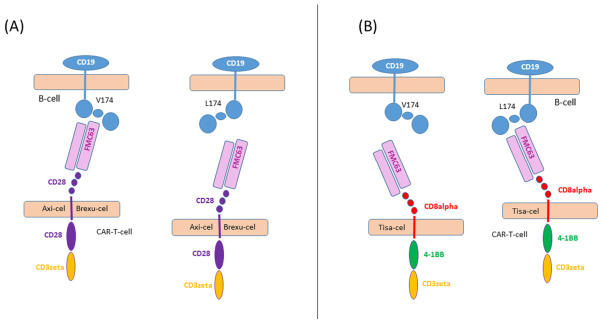
Schematic presentation of the two common variants of the CD19 antigen encoded by single-nucleotide polymorphism (SNP) rs2904880 encoding V174 and L174 (blue). The anti-CD19-FMC63 (pink) in CAR-T cell products axi-cel and brexu-cel may exhibit a greater binding affinity to the CD19 V174 variant and reduced affinity to the CD19 L174 variant (**A**). The anti-CD19-FMC63 in tisa-cel may exhibit a greater binding affinity to the CD19 L174 variant and reduced affinity to the CD19 V174 variant (**B**). The differential binding affinities are hypothetical.

The genotype-specific associations observed in our MCL cohort and in DLBCL [26] suggest that rs2904880 may not only act as a prognostic marker, but could also mechanistically influence CD19-directed CAR-T cell responses. Recent clinical observations in DLBCL indicate that the V174 genotype is associated with an improved outcome under axi-cel, similar to our findings with brexu-cel in MCL, whereas the opposite pattern is seen under tisa-cel [33]. Building on structural modeling and functional assays, the authors proposed that this divergence is driven by the distinct hinge regions of the CD19 CARs. These hinge regions differentially modulate the avidity and cytotoxic capacity of the CAR-T cells toward the CD19 V174 and L174 variants, such that the SNP rs2904880 subtly reshapes the CD19 epitope and thereby alters how efficiently each product can engage and eliminate malignant B-cell [35,36,37]. The depicted working model is hypothesis-generating and has not been validated (Figure 2).

Structural analyses of CD19 antibody complexes have shown that monoclonal antibodies can differ markedly in their affinity for CD19. These differences translate into distinct functional behavior of the corresponding CD19 CAR constructs. Specific CD19 variants such as Arg163Leu and Leu174Val, as well as the deletions affecting exons 1–3, have been mapped to key interaction sites and linked to impaired binding, loss of cytotoxicity and clinical immune escape with relapse under the FMC63-based CD19 CAR-T cell therapy, demonstrating that even subtle antigen variants can substantially alter CAR engagement [38,39,40]. These observations support the concept that both CAR design and *CD19* sequence variation can generate variant-specific affinity differences, supporting the plausibility of our working model that the germline polymorphism rs2904880 may modulate the interaction of individual CD19 CAR products. From a clinical perspective, brexu-cel is an established treatment option for patients with r/r MCL, yet the robust biomarkers that can predict the long-term benefit or early treatment failure are still lacking. Our data suggest that *CD19* rs2904880 may modestly contribute to outcome heterogeneity after CD19-directed CAR-T cell therapy, raising the possibility that the germline variation in the target antigen could, if validated, become a component of future risk stratification approaches. In principle, such a marker might help refine post-infusion surveillance strategies or inform an early consideration of an additional intervention in patients at an increased risk of poor outcomes, but at present this remains a conceptual perspective, rather than a clinically actionable strategy. Future implementation would require validation in large, prospectively collected multicenter cohorts, ideally including several hundred brexu-cel treated MCL patients and the integration of *CD19* rs2904880 into multivariable models alongside established clinical and molecular risk factors. Only if the polymorphism provides reproducible, independent prognostic information and improves the performance of such composite risk scores would its use in routine risk stratification be justified.

Several features of our study constrain the clinical applicability of these findings. The analysis was retrospective and single-center with a limited sample size and follow-up time, with the associated shortness of statistical power and likelihood of chance associations, particularly for subgroup and survival analyses. The discrepancy between the Gehan–Breslow–Wilcoxon and log-rank tests for the overall survival and the absence of a significant effect on the progression-free survival further underline the exploratory nature of the observed genotype signal. Moreover, the incomplete molecular characterization and the potential for residual confounding by unmeasured disease- or treatment-related factors cannot be excluded. Taken together, these limitations argue that *CD19* rs2904880 should currently be regarded as a hypothesis-generating candidate rather than as a clinical decision-making tool, and *CD19* genotyping should not influence the indication for brexu-cel CAR-T cell therapy outside of research settings.

Overall, our findings indicate that the *CD19* rs2904880 polymorphism is not associated with MCL disease susceptibility but may exert a modest, genotype-dependent influence on overall survival after brexu-cel CAR-T cell therapy. The lack of a consistent effect on progression-free survival and the limited robustness of the survival analyses underscore the exploratory nature and required confirmation. Future studies should aim to validate these observations in larger, ideally multicenter and prospectively collected cohorts with harmonized use of CD19-directed CAR-T cell products and comprehensive molecular characterization. The integration of *CD19* rs2904880 into broader models that combine clinical, disease-intrinsic and host-related factors may help to clarify whether this polymorphism has reproducible prognostic or even predictive value across different treatment settings. Ultimately, mechanistic work will be needed to delineate how *CD19* germline variation can influence CAR-T cell engagement and function and whether genotype-informed strategies can translate into improved patient outcomes.

## 5. Conclusions

*CD19* rs2904880 may exert a modest, genotype-dependent influence on overall survival after brexu-cel CAR-T cell therapy in relapsed or refractory mantle cell lymphoma. Notably, the direction of the association between CD19 rs2904880 and outcome in our MCL cohort appears inverse to prior reports in DLBCL and also varies across different CD19 CAR-T cell products, indicating that the impact of this polymorphism is highly context-dependent. Although this association is exploratory and based on a limited cohort, it highlights germline variation in the target antigen as a potential contributor to outcome heterogeneity after CD19-directed CAR-T cell therapy. Larger, prospective multicenter studies are needed to validate these findings and to clarify whether *CD19* polymorphism should be incorporated into future risk stratification strategies in MCL immunotherapy.

## Figures and Tables

**Table 1:** Baseline Clinical Characteristics.

		rs2904880		
Parameter	Total *n* = 21	V174hom *n* = 13	L174Vhet *n* = 8	*p*-Value
Male to female ratio	15:6 (2.5)	9:4 (2.3)	6:2 (3)	0.99
Age at ID, years, median (range)	67 (42–80)	68 (42–76)	62 (48–80)	0.90
Cytomorphological diagnosis				0.44
classic subtype, *n* (%)	14 (67)	7 (54)	7 (87)	
blastoid subtype, *n* (%)	6 (28)	5 (38)	1 (13)	
pleomorphic subtype, *n* (%)	1 (5)	1 (8)	0	
Stage at ID				0.65
I, *n* (%)	1 (5)	0	1 (13)	
II, *n* (%)	2 (10)	1 (8)	1 (13)	
III, *n* (%)	1 (5)	1 (8)	0	
IV, *n* (%)	16 (75)	11 (84)	5 (62)	
nd, *n* (%)	1 (5)	0	1 (13)	
MIPI				0.99
Low risk (5.1–5.5)	2 (10)	1 (8)	1 (12)	
Intermediate risk (5.7–6.5)	14 (66)	9 (69)	5 (63)	
High risk (6.7–9.1)	5 (24)	3 (23)	2 (25)	
Prior lines of treatment				0.55
1, *n* (%)	2 (10)	2 (15)	0	
2, *n* (%)	9 (43)	6 (46)	3 (43)	
≥3, *n* (%)	10 (48)	5 (38)	5 (48)	
Previous radiotherapy	4 (19)	1 (8)	3 (38)	0.25
Previous SCT	8 (38)	5 (38)	3 (38)	0.99
CNS involvement	3 (14)	3 (23)	0	0.26

Percentages are column-based and refer to the respective group totals; ID: initial diagnosis; CNS: central nervous system; MIPI: Mantle Cell Lymphoma International Prognostic Index; SCT: stem-cell transplantation.

**Table 2:** Treatment characteristics.

		rs2904880		
Parameter	Total *n* = 21	V174hom *n* = 13	L174Vhet *n* = 8	*p*-Value
Age at CAR-T, years, median (range)	73 (56–82)	70 (56–82)	74 (57–81)	0.66
Time ID to CAR-T, years, median (range)	4 (1–24)	4 (1–14)	5 (1–24)	0.71
Remission Status pre CAR-T				0.24
CR, *n* (%)	3 (14)	3 (23)	0 (0)	
PR, *n* (%)	3 (14)	1 (8)	2 (25)	
SD, *n* (%)	8 (38)	6 (46)	2 (25)	
PD, *n* (%)	7 (33)	3 (23)	4 (50)	
Bridging chemotherapy, *n* (%)	10 (48)	6 (54)	5 (38)	0.66
Bridging radiotherapy, *n* (%)	1 (5)	1 (8)	0 (0)	0.99
LDH pre CAR-T (U/L), median (range)	210 (126–1359)	191 (126–374)	297 (181–1359)	0.08
Elevated LDH (>250 U/L), *n* (%)	8 (42)	4 (33)	4 (57)	0.38

Percentages are column-based and refer to the respective group totals; ID: initial diagnosis; CR: complete response; PR: partial response; SD: stable disease; PD: progressive disease; LDH: lactate dehydrogenase.

**Table 3:** Early therapy-associated toxicities.

		rs2904880		
Parameter	Total *n* = 21	V174hom *n* = 13	L174Vhet *n* = 8	*p*-Value
CRS, *n* (%)	16 (76)	11 (85)	5 (63)	0.33
Grade 1	8 (38)	6 (46)	2 (25)	
Grade 2	6 (29)	5 (38)	1 (13)	
Grade 3	1 (5)	0 (0)	1 (13)	
nd	1 (5)	0 (0)	1 (13)	
Time to CRS, days, median (range)	5 (1–11)	5 (1–8)	5 (1–11)	0.64
ICANS, *n* (%)	10 (48)	6 (46)	4 (50)	0.99
Grade 1	6 (29)	3 (23)	3 (38)	
Grade 2	0 (0)	0 (0)	0 (0)	
Grade 3	3 (14)	2 (15)	1 (13)	
Grade 4	1 (5)	1 (8)	0 (0)	
Time to ICANS, days, median (range)	10 (6–18)	11 (6–18)	10 (7–11)	0.72
CAR-T copies per μg cfDNA, median (range)	7237 (0–92,877)	9514 (76–92,877)	6758 (0–31,568)	0.75
Time to peak CAR-T, days, median (range)	13 (6–192)	13 (6–192)	13 (7–26)	0.99
CRP peak, mg/L, median (range)	56 (3–276)	37 (3–103)	86 (29–276)	0.05
Time to CRP peak, days, median (range)	6 (0–28)	6 (0–12)	9 (1–28)	0.23
IL-6 peak, pg/mL, median (range)	638 (7–50,000)	1282 (7–50,000)	435 (43–5410)	0.50
Time to IL-6 peak, days, median (range)	8 (3–35)	7 (3–18)	12 (6–35)	0.09
Ferritin peak, μg/L, median (range)	1474 (135–16,920)	1617 (135–16,920)	1429 (225–5239)	0.99
Time to ferritin peak, days, median (range)	11 (1–32)	11 (1–32)	12 (1–31)	0.51

Percentages are column-based and refer to the respective group totals; CRS: cytokine release syndrome; ICANS: Immune Effector Cell-associated Neurotoxicity Syndrome; CRP: C-reactive protein; IL-6: Interleukin-6.

**Table 4:** Treatment outcomes after CAR-T cell therapy.

		rs2904880		
Parameter	Total *n* = 21	V174hom *n*= 13	L174Vhet *n* = 8	*p*-Value
Best response OR *n* (%)	19 (90)	13 (100)	6 (75)	0.24
CR, *n* (%)	16 (76)	11 (85)	5 (63)	
PR, *n* (%)	3 (14)	2 (15)	1 (13)	
SD, *n* (%)	0 (0)	0 (0)	0 (0)	
PD, *n* (%)	2 (10)	0 (0)	2 (25)	
CR at last follow up, *n* (%)	13 (62)	9 (69)	4 (50)	0.65
Lymphoma-related event, *n* (%)	4 (19)	2 (15)	2 (25)	0.62
Relapse after achieving CR/PR	3 (14)	2 (15)	1 (13)	
Primary refractory disease	1 (5)	0 (0)	1 (13)	
Death, *n* (%)	9 (43)	4 (31)	5 (63)	0.20
Tumor-associated	4 (19)	2 (15)	2 (25)	
Therapy-associated	2 (10)	1 (8)	1 (13)	
Secondary malignancy	1 (5)	1 (8)	0 (0)	
Other/Unknown	2 (10)	0 (0)	2 (25)	

Percentages are column-based and refer to the respective group totals; OR: overall response; CR: complete response; PR: partial response; SD: stable disease; PD: progressive disease.

**Table 5:** Clinical outcome after CAR-T cell therapy, multivariate analysis.

	PFS		OS	
Predictors	HR (95% CI)	*p*-Value	HR (95% CI)	*p*-Value
CD19 V174hom vs. V174Lhet	0.19 (0.03, 1.3)	0.10	0.08 (0.01, 0.8)	0.04
LDH high vs. normal	39 (2, 630)	0.01	63 (8, 1560)	0.01
Prior therapy lines ≥3 vs. <3	6.2 (0.7, 55)	0.10	5.8 (0.5, 64)	0.15
CRS vs. no CRS	1.6 (0.2, 15.3)	0.69	2.5 (0.2, 28.5)	0.48

CRS: cytokine release syndrome; HR: hazard ratio.

## Data Availability

The data presented in this study are available on request from the corresponding author.
